# Accurate prediction of RNA-binding protein residues with two discriminative structural descriptors

**DOI:** 10.1186/s12859-016-1110-x

**Published:** 2016-06-07

**Authors:** Meijian Sun, Xia Wang, Chuanxin Zou, Zenghui He, Wei Liu, Honglin Li

**Affiliations:** State Key Laboratory of Bioreactor Engineering, Shanghai Key Laboratory of New Drug Design, School of Pharmacy, East China University of Science and Technology, 130 Mei Long Road, Shanghai, 200237 China

**Keywords:** Protein-RNA interactions, Residue triplet interface propensity, Residue electrostatic surface potential, Random forest classifier, Structural analysis

## Abstract

**Background:**

RNA-binding proteins participate in many important biological processes concerning RNA-mediated gene regulation, and several computational methods have been recently developed to predict the protein-RNA interactions of RNA-binding proteins. Newly developed discriminative descriptors will help to improve the prediction accuracy of these prediction methods and provide further meaningful information for researchers.

**Results:**

In this work, we designed two structural features (residue electrostatic surface potential and triplet interface propensity) and according to the statistical and structural analysis of protein-RNA complexes, the two features were powerful for identifying RNA-binding protein residues. Using these two features and other excellent structure- and sequence-based features, a random forest classifier was constructed to predict RNA-binding residues. The area under the receiver operating characteristic curve (AUC) of five-fold cross-validation for our method on training set RBP195 was 0.900, and when applied to the test set RBP68, the prediction accuracy (ACC) was 0.868, and the F-score was 0.631.

**Conclusions:**

The good prediction performance of our method revealed that the two newly designed descriptors could be discriminative for inferring protein residues interacting with RNAs. To facilitate the use of our method, a web-server called RNAProSite, which implements the proposed method, was constructed and is freely available at http://lilab.ecust.edu.cn/NABind.

**Electronic supplementary material:**

The online version of this article (doi:10.1186/s12859-016-1110-x) contains supplementary material, which is available to authorized users.

## Background

Protein-RNA interactions play a vital role in various fundamental cellular processes, such as transcription and the post-transcriptional processing of pre-mRNA, the stability and localization of mRNA and translation [1]. Defects in these RNA-binding proteins (RBPs) may lead to many human diseases, including neuropathies, muscular atrophies and cancer [2]. Consequently, the detection of the RNA-binding residues (RBRs) in a protein will provide insight into the underlying molecular mechanism of these important biological processes and contribute to the development of new therapeutic methods for relevant diseases. Several experimental approaches are used to detect RNA-binding sites in a protein, such as X-ray crystallography, nuclear magnetic resonance (NMR), ultraviolet crosslinking and immunoprecipitation (CLIP) [3, 4] and site-directed mutagenesis. However, these experimental methods are inefficient in identifying RBRs because they involve laborious and time-consuming procedures [5, 6]; therefore, accurate and efficient computational techniques are required to infer the most likely candidate residues in RNA interfaces directly from the sequences or/and structures of RBPs. With the assistance of these excellent computational methods, researchers can perform more targeted assays to detect RNA-binding sites and further explore the mechanisms behind the interactions between proteins and RNAs.

Recently, a significant number of computational methods predicting RNA-binding sites have been developed through the comprehensive analysis of sequences and structures of RNA-binding proteins. Several fundamental structural and physicochemical principles underlying the mutual recognition of protein and RNA have been discovered [7–13]. These computational predictors can be broadly divided into sequence- and structure-based predictors in terms of the key information that they use to characterize protein residues. Sequence-based methods are usually machine learning-based and their classifiers are trained using features derived directly from protein sequences. Amongst the sequence-derived features, evolutionary information in the form of position-specific scoring matrix (PSSM) is one of the most frequently used features and is proved a powerful descriptor for discriminating RBRs from non-RBRs [14–19]. Several other descriptors are also commonly used including predicted solvent accessibility [20–22], predicted secondary structure [22], physicochemical property [18, 20, 21, 23, 24]. Most of these sequence-based methods are developed by support vector machine (SVM), but in a few methods, some other classification algorithms are adopted, such as Naïve Bayes [25], C4.5 decision tree [18]. Unlike sequence-based methods, structure-based methods use various features extracted from the atomic coordinates of protein-RNA complexes to recognize interface residues using different techniques, such as machine learning [26–31], scoring [32–36] and template-based methods [37, 38]. As proteins directly recognize their target RNAs by some of their surface residues and because the geometrical properties of these surface residues may be different from those not in the protein-RNA interfaces, several structure-based approaches have calculated surface geometry from the structure of an RBP [32, 39]. In addition, various other structural features, such as solvent-accessible surface, electrostatics and secondary structure, as well as evolutionary and statistical features are also frequently used. Template-based methods commonly align a target protein structure to the known protein-RNA complexes in the templates library using a certain structural alignment program [38, 40] and then select a most likely predicted complex structure containing the target protein and RNA of the template library. Finally, the RNA-binding residues of the protein are inferred directly from the predicted complex structures. One recently developed structure-based method could predict both RNA- and DNA-binding residues with excellent performance [36].

The number of known proteins that can interact with RNA only account for a small fraction of the solved structures, other structures may also have unknown potential RNA-binding activities. Structure-based prediction methods can use the known structural information to identify likely RNA-binding sites on the structures of hidden RBPs, and these discriminative structural characteristics cannot be calculated from pure protein sequences because the mechanisms of protein folding from sequence to spatial structure are not exactly known. Consequently, the key to accurately predicting RBRs from protein structures is to compute structural descriptors that can distinguish between residues that interact with RNA and those that cannot interact with RNA efficiently.

To design structural features with relatively strong discriminatory power and excellent applicability, two structural features are computed, the residue electrostatic surface potential and the triplet interface propensity. Although the calculation of the residue electrostatic surface potential in our study only involved several simple processes without considering other factors such as solvent and ion, this newly designed feature was helpful for detecting RBRs, and the electrostatic interactions between a protein and its partner RNA are commonly observed; therefore, the electrostatic feature was expected to be applicable to different RBPs. The feature triplet interface propensity in our study was calculated based on the protein secondary structure and spatial atomic coordinate information in each protein-RNA complex; moreover, for each interface triplet type, we divided the interface triplet into four subtypes according to the RNA-binding properties of two neighboring residues of the centre residue. We believe that the incorporation of different types of features may uncover the mechanisms for protein-RNA interaction from different angles and will help a classifier generate a more accurate prediction. Therefore, several excellent features, such as evolutionary information in the form of PSSMs, physicochemical properties and geometrical features were used together with the two newly designed structural features. To encode a target protein residue with the feature information of its neighboring residues, we searched the optimal type and size of a patch containing several neighboring residues of the target residue for each type of feature. Thus, we developed a random forest classifier, as was implemented in a web server named RNAProSite (see ‘Methods’ section), combined with hybrid features from both sequences and structures, and the area under the receiver operating characteristic (ROC) curve (AUC) of five-fold cross-validation on a non-redundant training dataset containing 195 RBP chains was 0.900.

## Methods

### Datasets

Two groups of datasets are used in this study: i) RBP195 was used to construct the prediction model proposed in this study; RBP68 was used for benchmark test of our prediction model with other common available models. ii) RBP138 and RBP42 were constructed for evaluating the importance of some important factors on the prediction performance such as the composition of datasets, the selection of machine-learning algorithms and the definition methods of RNA-binding sites of proteins.

### RBP195 and RBP68

All of the available structures of protein-RNA complexes in the PDB ≤ 3.0 Å and resolved by X-ray crystallography before January 2014 were obtained. Then, we used the PISCES program [41] to ensure that the resulting dataset shared ≤ 40 % sequence similarity. Thus, 308 RNA-binding protein chains were left, and two of which (chains 1 and 3 of protein 2ZJR in PDB) had residues lacking carbon alpha (C_α_) atoms; therefore, we discarded the two chains to enable the execution of the DSSP [42] software, which was used to generate secondary structure features for proteins. Then, we excluded protein chains whose sequence length was less than 4, and finally, a dataset consisting of 263 protein chains was constructed, 195 of which were randomly selected to constitute the RBP195 for model training, the others were used to construct RBP68 for the benchmark of prediction models. The ratio of RBRs and non-RBR from RBP195 and RBP68 is about 5.73 and 5.29.

### RBP138 and RBP42

As the sequence of identity of 40 % cannot necessary exclude redundancy, so we used the cutoff of 25 % for PISCES program [41] to remove the redundant sequences in RBP195 and RBP68, and Many RNA binding proteins in different organisms may share sequence identity below 20 % but with the similar structure, and such homologous proteins could easily be detected by PSSM profile, so we further removed the protein chains sharing the same class, architecture, topology and homologous (CATH) [43] code with other protein chains. Finally, a dataset RBP138 containing 138 RBP chains was constructed from RBP195, and RBP42 containing 42 RBP chains was derived from RBP68. No pair of chains in (or between) RBP138 and RBP42 shared more than 25 % sequence similarity and same CATH code. The ratio of non-RBRs and RBRs in RBP138 and RBP42 is about 9.69 and 10.08. A complete list of all of the PDB codes for the datasets constructed could be found in Additional file 1.

Distance-based definition of RNA-binding residues is frequently used [30, 33, 35, 36]. Two kinds of cut-off values for the definition of RNA-binding protein residues, namely 5 Å and 3.5 Å, were used in this study to explore the effects of the selection of cut-off values on the prediction accuracy of our method. A cut-off value of 5 Å was used to define the RNA-binding sites on RBP195 and RBP68; specifically, an amino acid residue was considered an RNA-contacting residue if it contained one or more heavy atoms within 5 Å of any atom in the bound RNA. The cut-off value of 3.5 Å was used on RBP138 and RBP42.

### Random forest (RF) implementation

The prediction of RBRs is actually a binary classification problem, and RF was used to perform the binary classification in this study. The RF algorithm is a popular machine-learning method that uses an ensemble of tree-structured classifiers [44], each of the tree classifiers in the forest is constructed using different bootstrap samples from the original training data set. The RF is very user friendly because it is usually not sensitive to its only two main parameters (the number of variables in the random subset at each node and the number of trees in the forest) [45], which makes RF more efficient than the frequently used SVM because learning with SVM is time-consuming with respect to the selection of the optimal parameters and kernel functions for the classifier. In addition, RF is relatively robust to outliers and noise. Several practical applications of RF have demonstrated excellent performance in prediction studies [26, 46, 47]. An open-source RF tool for the MATLAB windows (available at http://code.google.com/p/randomforest-matlab/) was used to develop our classifier, in which the default parameters for RF were used.

### Protein features

To develop a powerful structure-based site predictor for RBPs, one of the keys is to design discriminative features derived from the protein structure information and to adopt other different features charactering the mechanisms of protein-RNA interactions. In this study, five types of features were used to characterize protein residues: two newly designed structure-derived features (electrostatic feature and triplet interface propensity) and three other common excellent features (PSSM profile, geometrical characteristic and physicochemical property).

### Electrostatic feature for each surface residue

Protein surfaces mediating protein-RNA interactions are commonly characterized by positive electrostatic potential due to the charge complementarity with negatively charged phosphate groups in the RNA [11, 12]. Moreover, these surface residues are commonly spatially near to each other, therefore, we calculated the electrostatic potential value for each residue located on the protein surface and then applied a density-based clustering algorithm to determine whether an amino acid residue is in the largest surface patch with positive electrostatic potential and negative electrostatic potential. Although the role of electrostatic interactions has been extensively used, we provided a new procedure for the calculation of electrostatic interactions in this study.

First, the DMS program (available at http://www.cgl.ucsf.edu/Overview/software.html#dms) was used to generate the surface points of each RBP extracted from protein-RNA complexes. The output consists of a series of atoms and surface point records; each atom is followed by the surface points that belong to it. Second, each protein structure was assigned charge and radius parameters from the PARSE force field [48] using the PDB2PQR software [49], which could also rebuild the missing heavy atoms of the initial protein structure and then add hydrogen atoms to the reconstructed structure to ensure the calculation accuracy of electrostatic potential. Third, we calculated the electrostatic potential at a surface point; the classical formula for the calculation is given by:

1 $$ {V}_F={\displaystyle \sum_i\frac{q_i}{\varepsilon \left|{r}_i-F\right|}} $$

Where *q*_*i*_ is the charge for atom *i* whose Euclidean distance away from point *F* is |*r*_*i*_ − *F*|. Here, we used a distance-dependent dielectric constant to define *ε* as |*r*_*i*_ − *F*|. The calculation of electrostatic potential *V*_*F*_ considers all of the atoms within a distance threshold of 7 Å as distances ≤7 Å can be important for protein-nucleic acid interactions [50]. The electrostatic potential for an atom (*V*_*a*_) is defined as the mean of *V*_*F*_ values of all of the surface points belonging to the atom. Similarly to the calculation for an atom, a residue’s electrostatic surface potential (*V*_*r*_) is defined as the mean of *V*_*a*_ values of its component atoms. For any residue that has no surface points according to the results of the DMS program, its *V*_*r*_ value is assigned as zero.

To construct the largest spatially continuous positive patch on the RBP surface, DBSCAN [51], a density-based spatial clustering algorithm, was used to find the largest positive surface patch and the largest negative surface patch on a protein. We initially represent a surface amino acid residue as a point, and the *x*, *y* and *z* for the point are calculated as follows:

2 $$ x={\displaystyle {\sum}_i\Big({x}_i\times {N}_i/N}\Big), $$

3 $$ y={\displaystyle {\sum}_i\Big({y}_i\times {N}_i/N}\Big), $$

4 $$ z={\displaystyle {\sum}_i\Big({z}_i\times {N}_i/N}\Big) $$

where (*x*, *y*, *z*) is the coordinate of the point representing a surface residue, (*x*_*i*_, *y*_*i*_, *z*_*i*_) is the coordinate of atom *i* of the surface residue, *N*_*i*_ is the number of surface points belonging to an atom *i* of the surface residue, and *N* is the sum of surface points belonging to all of the atoms of the surface residue. Based on a set of coordinates of the points representing protein residues, DBSCAN [51], a density-based spatial clustering algorithm was used to cluster the residues with positive *V*_*r*_ values to construct the largest positive surface patch or with negative *V*_*r*_ values to construct the largest negative surface patch. The reason for using DBSCAN instead of other clustering methods, such as hierarchical clustering, which has been used in several studies [8, 52], is because the protein-RNA interfaces frequently have irregular shapes and DBSCAN can find arbitrarily shaped clusters on the protein surface. Two parameters are required by DBSCAN: the minimum number of points (*minPts*) needed to form a cluster and *ε*. The clustering algorithm can find all of the potential clusters that consist of a maximum of possible core points and their neighboring points within a sphere of radius *ε*. A core point is defined as a point surrounded by no less than *minPts* neighboring points within a distance *ε*. All of the core points in a cluster must satisfy one condition: for each two core points, represented by *x* and *y*, there exists at least one consecutive sequence of *n* + 2(*n* ≥ 1) core points represented by [*x*, *p*_1_, …, *p*_*i*_, …, *p*_*n*_, *y*] in the cluster and each core point *p*_*i*_(1 ≤ *i* ≤ *n*) is not farther away from its next and former core point in the sequence than a given distance *ε*. Based on the clustered surface residues, we selected the cluster containing the largest number of residues to be the largest surface patch. As the distances of important interactions (interactions of hydrogen bonds, stacking interactions, van der Walls interactions, electrostatic interactions, hydrophobic interactions, etc.) between proteins and RNAs are usually ≤7 Å [10], so *ε* was set to 7 Å. According to our statistics, there averagely exist about two surface residues with negative (or positive) electrostatic potential within a distance of 7 Å of a surface residue with negative (or positive) surface electrostatic potential in RBP195, thus *minPts* was set to 3, which is larger than the average value 2. Finally, the electrostatic feature for a particular residue in a protein sequence can be described by a three-dimensional vector, the first value in the vector is the *V*_*r*_ of the residue; the second is assigned by number 1 or 0 to specify whether the residue is in the largest positive patch; and the third is assigned by number 1 or 0 to specify whether the residue is in the largest negative patch. For residues with no surface points, the three values are assigned to the number 0.

### Triplet interface propensity

The sequentially adjacent neighbors of interface RNA-binding residues have significant biases in amino acid types [25], this phenomenon also exists in protein-DNA interfaces [52]. Here, we designed a statistical feature to describe the phenomenon, namely triplet interface propensity, based on the RBP chains in the datasets used here. A consecutive three-residue segment along the sequence of an RBP chain is designated as an interface triplet when its centre residue is RNA-binding and the three-residue segment is a surface triplet, in which each residue has a relative solvent accessibility (RSA) greater than 3 % (roughly determined by prediction performance when different RSA cutoff values were selected, seen in Additional file 2). The calculation of triplet interface propensity is first defined by the following equation:

5 $$ {R}_x={\displaystyle \sum_{p=1}^{p=n}\left({f}_{x,p}\times \ln \frac{f_{x,p}}{f_{x\hbox{'},p}}\right)} $$

Where *x* represents a type of triplet on protein-RNA interfaces, *x* ' represents the same surface triplet as *x* (all of the three residues in *x* ' are with RSA greater than 3 % and are the same as *x* in the way of composition and arrangement, but not necessarily on protein-RNA interfaces), *p* represents a certain RBP chain, *n* is the number of protein chains involved in the statistical procedure, *f*_*x*,*p*_ represents the frequency of an interface triplet *x* in the interfaces of a RBP chain *p* and its bound RNA, *f*_*x*,*p*_ is calculated as:

6 $$ {f}_{x,p}={N}_x/{N}_{all} $$

Where *N*_*x*_ represents the number of heavy atoms interacting with RNA in the triplet *x*, and *N*_*all*_ is the total number of heavy atoms interacting with RNA in protein *p. f*_*x* ',*p*_ represents the frequency of a surface triplet *x* ' in an entire protein *p*, *f*_*x* ',*p*_ is calculated as:

7 $$ {f}_{x\hbox{'},p}={T}_{x\hbox{'}}/{T}_{all} $$

where *T*_*x* '_ represents the number of surface triplets of *x* ' in protein *p* and *T*_*all*_ represents the number of all of the likely surface triplets in protein *p*. An interface triplet may have different types when considering the RNA-binding properties of the two neighboring residues of the centre residue. To specify the contributions of the two neighboring residues to the propensity of an interface triplet, we further described the propensity of a given interface triplet *x* with a vector of length 4. Specifically, *R*_*x*_ is represented by *R*_*x*1_, *R*_*x*2_, *R*_*x*3_ and *R*_*x*4_; these four values describe the propensities of four subtypes of an interface triplet, namely, triplet_1, triplet_2, triplet_3 and triplet_4, respectively. In triplet_1 of interface triplet, both the first and third residues are RNA-binding, whereas in triplet_4, the first and third residues are not RNA-binding. In triplet_2, the first residue is RNA-binding, and the third is not; however, in triplet_3, the third residue is RNA-binding, and the first is not. Therefore, any interface triplet may have one or more of the four subtypes, represented by triplet_1, triplet_2, triplet_3 and triplet_4.

The protein secondary structure information is widely used in the prediction of RNA-binding sites; here, we calculated the propensities of secondary structure types for interface triplets, and the secondary structure type of an interface triplet was determined by that of its centre residue. The DSSP program [42] was used to calculate the secondary structure type for each individual amino acid residue, and the resulting eight secondary structure types were further divided into three states using the following rule: secondary structure types I, G, and H were considered to be helices and represented by the number 1; types E and B were considered to be sheets and represented by the number 2; and the other types were considered to be coils and represented by the number 3. The following equation was used to calculate the propensity for the secondary structure type of an interface triplet:

8 $$ {I}_{x,s}=\left({\displaystyle \sum_{p=1}^{p=n}{N}_{x,s,p}}\right)/\left({\displaystyle \sum_{p=1}^{p=n}{\displaystyle \sum_{s^{\hbox{'}}=1}^{s^{\hbox{'}}=3}{N}_{x^{\hbox{'}},{s}^{\hbox{'}},p}}}\right) $$

Where *x* represents a type of triplet in the protein-RNA interface, *x* ' represents the same triplet as *x* on the protein surface, *N*_x,s,p_ represents the number of interface triplets *x* with secondary structure type *s* in protein *p*, and *N*_*x* ',*s* ',*p*_ is the number of existing surface triplets *x*’ with secondary structure type *s*’.

When the propensity for the secondary structure type of an interface triplet is considered, the 4D vector describing the propensity for a supposed interface triplet *x* should be calculated using two procedures. One procedure is to compute the values of *R*_*x*1,_*R*_*x*2,_ *R*_*x*3,_ *R*_*x*4_ and *I*_*x*,*s*_ for the triplet *x*, whose secondary structure type is known as *s* according to the output of DSSP program [42], after which the 4D vector can be finally defined as $\left({I}_{x,s}\times {R}_{x1},\kern0.5em {I}_{x,s}\times {R}_{x1},\kern1em {I}_{x,s}\times {R}_{x3},\kern0.5em {I}_{x,s}\times {R}_{x4}\right)$.

### PSSM profile

The position-specific scoring matrix (PSSM), generated by using the PSI-Blast program [53] to search against the NCBI’s non-redundant (NR, released on 14 May 2011) database (the iteration time was set to 3 and *E*-value cutoff to 0.001), was used to represent the evolutionary conservation of each amino acid in a protein sequence. For those with missing residues in protein structures, we just use its sequence to generate PSSM profile and then we remove the information of missing residues from the generated PSSM profile. The generated PSSM scoring matrix of a protein with *N* residues has 20 × *N* elements.

### Geometrical characteristic

In this study, the accessibility of protein residues was calculated using the program NACCESS [54], which uses the Lee and Richards algorithm [55]. Five values that describe the relative solvent accessibility (RSA) of all atoms, side chain atoms, main chain atoms, non-polar side chain atoms and polar side chain atoms of each amino acid residue were extracted from the outputs. To characterize the shape feature of a residue on the protein surface, the *CX* value [56] of the residue is calculated by the summation of the *CX* values of its component atoms. For a residue with one or more atoms with an accessible surface area (ASA) that exceeds 1.0 Å^2^, the residue may have one of the following shape characteristics based on the _*CX*_ value of the residue: dented (*CX* < −0.5) represented by the number 0, intermediate (−0.5 ≤ *CX* ≤ 0.5) represented by the number 1 and protruded (*CX* > 0.5) represented by the number 2. For a residue that is buried and has no _*CX*_ value, its shape characteristic is set to the number 3. The ASA for each atom was also calculated by the program NACCESS. The RSA and shape characteristics for a residue were considered the geometrical characteristics of the residue as represented by a 6D vector (five types of RSA values and one _*CX*_ value).

### Physicochemical property

In our study, the physicochemical property of a residue was characterized by a vector of length 10, representing the ten types of properties of an individual amino acid residue extracted from the AAIndex [57], as shown in Table 1. The selection of properties from AAIndex mainly involved the calculation of correlation coefficient between interface propensities (*P*_*k*_) [34] and properties of twenty amino acid residues in AAIndex. The details could be seen in Additional file 3.

**Table 1:** List of the AAIndex indices used in this article

AAIndex ID	Description
FINA910101 [60]	Helix initiation parameter at position i-1 (Finkelstein et al., 1991)
OOBM850101 [61]	Optimized beta-structure-coil equilibrium constant (Oobatake et al., 1985)
TANS770108 [62]	Normalized frequency of zeta R (Tanaka-Scheraga, 1977)
TANS770106 [62]	Normalized frequency of chain reversal D (Tanaka-Scheraga, 1977)
WOEC730101 [63]	Polar requirement (Woese, 1973)
LEWP710101 [64]	Frequency of occurrence in beta-bends (Lewis et al., 1971)
ISOY800105 [65]	Normalized relative frequency of bend S (Isogai et al., 1980)
FAUJ880108 [66]	Localized electrical effect (Fauchere et al., 1988)
RICJ880105 [67]	Relative preference value at N2 (Richardson-Richardson, 1988)
COSI940101 [68]	Electron-ion interaction potential values (Cosic, 1994)

### Encoding scheme

Previous studies have demonstrated that considering the neighborhood of a residue can significantly improve the accuracy of identifying whether the residue is a RBR [26, 28]. Two types of patches to incorporate neighboring residues are commonly adopted: a sequential patch that is often used in sequence-based methods and a structural patch that is frequently employed in structure-based methods. A sequential patch of size *n* for a target residue is the set of *n*-1 residues nearest to the target residue along the primary protein sequence and the target residue itself. Similarly, a structural patch of size *n* for a target reside is defined as the set of the target residue and its *n*-1 nearest neighbor residues according to the Euclidean distance between the coordinate of these neighbor residues and that of the target residue [28]. In this study, we analyzed the prediction performance for each individual feature combined with the two types of neighborhood construction techniques, which was expected to select the best patch type with optimal size for a certain feature type. Then, for a single target residue that was initially represented by five types of descriptor vectors whose size are *d*_*1*_, *d*_*2*_, *d*_*3*_, *d*_*4*_ and *d*_*5*_ (feature vector size for electrostatic feature, triplet interface propensity, PSSM profile, geometrical characteristic, and physicochemical property, respectively) with optimal sizes of the optimal patch type for each descriptor of *s*_*1*_, *s*_*2*_, *s*_*3*_, *s*_*4*_ and *s*_*5*_ (the details for the selection of optimal patch type and patch size could be found in Additional file 4), respectively, the target residue is represented in a feature vector with 281 (∑_*i* = 1 : 5_(*d*_*i*_ × *S*_*i*_), *d*_*1*_ = 3; *d*_*2*_ = 4; *d*_*3*_ = 20; *d*_*4*_ = 6; *d*_*5*_ = 10; *s*_*1*_ = 11; *s*_*2*_ = 7; *s*_*3*_ = 5; *s*_*4*_ = 5; *s*_*5*_ = 9) elements.

### Evaluation measures for the prediction model

To assess the predictive power of RNAProSite on test datasets, five parameters were used, i.e., sensitivity (SN), specificity (SP), positive predictive value (PPV), accuracy (ACC), F-score and Matthews’s correlation coefficient (MCC). Mathematically, these parameters are defined in the following equations:

9 $$ SN=\frac{TP}{TP+FN}, $$

10 $$ SP=\frac{TN}{TN+FN}, $$

11 $$ PPV=\frac{TP}{TP+FP} $$

12 $$ ACC=\frac{TP+TN}{TP+TN+FP+FN}, $$

13 $$ MCC=\frac{TP\times TN\times FP\times FN}{\sqrt{\left(TP+FN\right)\left(TP+FP\right)\left(TN+FP\right)\left(TN+FN\right)}}, $$

14 $$ F- score=\frac{2\times TP}{2\times TP\times FP+FN}. $$

Where TP is the number of true positives, TN is the number of true negatives, FP is the number of false positives, and FN is the number of false negatives. The fivefold cross validation method was used to evaluate the prediction model. For fivefold cross validation, the entire protein residues in a dataset were randomly partitioned into five parts with approximately the same size, after which the classifier was trained in the four parts and tested on the remaining part. This procedure was repeated five times to ensure that each protein residues is tested once. The performance of fivefold cross-validation was measured by means of ROC curves and Area under the ROC Curve (AUC).

### Web server

The RNAProSite web server can be freely accessed at http://lilab.ecust.edu.cn/NABind.

## Results

### Distribution of electrostatic surface potentials

**Fig. 1:**
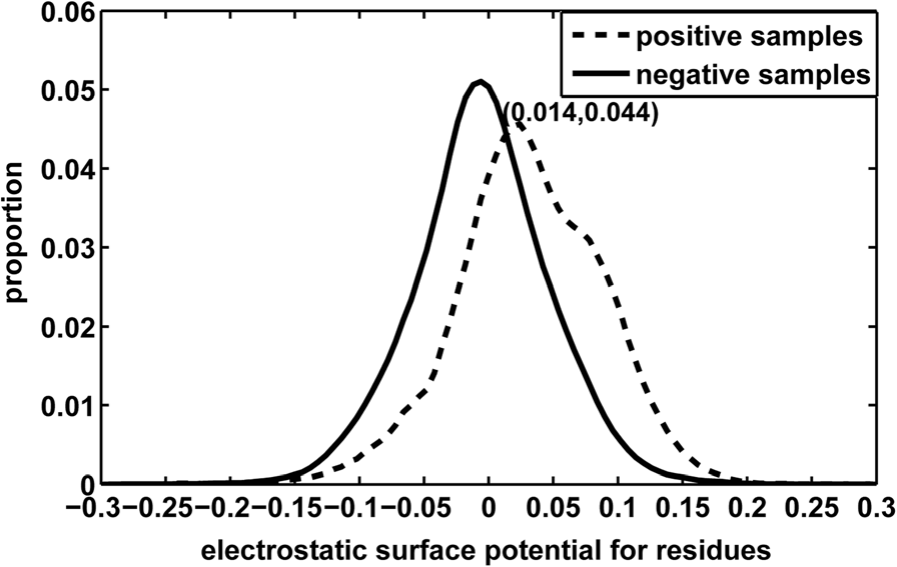
The distribution of electrostatic surface potentials for both positive (RNA-binding) and negative (non-RNA-binding) samples in RBP195, the cross point of the two distribution curves is at (0.014, 0.044)

To explain the importance of the electrostatic surface potential in the identification of RBRs, we analyzed the distribution of the electrostatic potential values for all of the RBRs and non-RBRs in RBP195 in Fig. 1. From the two distribution curves of positive samples and negative samples, the two distribution curves cross at a point whose electrostatic potential value is approximately 0.014, and when the electrostatic potential value is less than that of the cross point, the negative samples have a higher proportion than the positive samples, but the opposite occurs when the value is larger than that of the cross point. In total, the evident difference in the two distribution curves demonstrates that the tendency for a residue to be an RBR occurs when the residue has a positive electrostatic surface potential, whereas that for a residue to be a non-RBR occurs when the residue has a negative electrostatic surface potential.

### Distribution of clustered patches in RBRs and non-RBRs

**Fig. 2:**
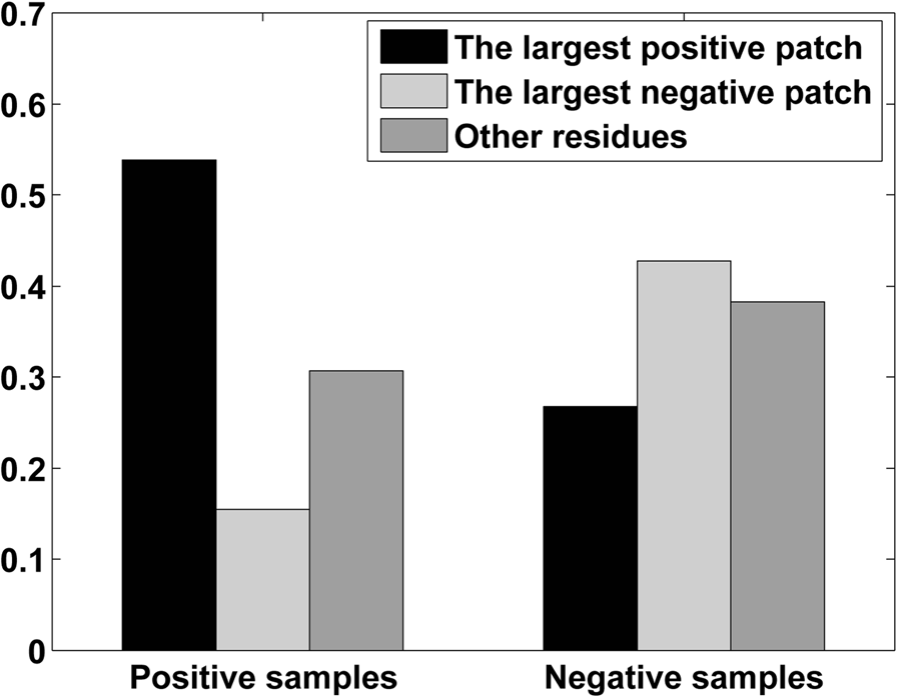
The distribution of patch types in positive samples and negative samples. The residues neither in the largest positive patch nor in the largest negative patch of each chain in RBP195 are labeled as “Other residues”

To investigate the effectiveness of the DBSCAN clustering that determines whether a surface residue is in the largest positive patch or largest negative patch, we counted all of the protein residues in RBP195 that interact and do not interact with RNA. For positive samples and negative samples in RBP195, the proportion of residues in the largest positive patch and largest negative patch of each protein chain was calculated. As shown in Fig. 2, the residues in the largest positive patch accounted for approximately 53.85 % of all of the positive samples, but the percentage of residues in the largest negative patch was only approximately 15.47 %, demonstrating the excellent capability of the clustering feature to distinguish residues involved in RNA-binding from those not involved and revealing the preference of interface residues for a connective surface area with positive electrostatic potential. A moderate percentage (30.68 %) of the residues belong to “Other residues”, indicating that some RNA-interacting residues were just not in the largest positive patch. When the composition of negative samples was analyzed, the residues in the largest negative patch accounted for approximately 42.72 % more than the percentage for those in the largest positive patch.

### Analysis of the triplet interface propensity and subtypes of interface triplet

**Fig. 3:**
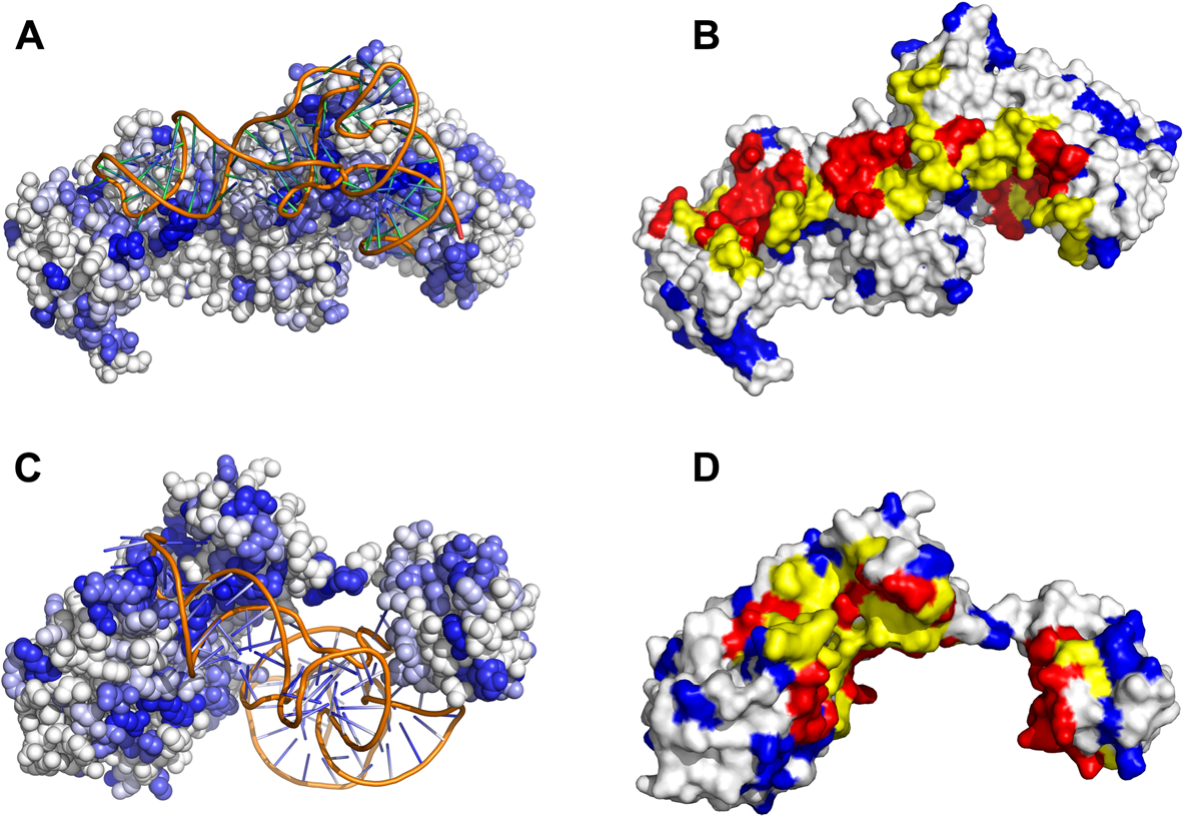
The triplet interface propensities for residues in protein 1QTQ_A (**a** and **b**) and 2ZZM_A (**c** and **d**). In A and C, the residues colored from white to blue (stands for propensity values from −0.0149 to 3.1023), and the darker the blue color of the residues, the more likely the residues are involved in RNA-protein interactions. In B and D, the residues having triplet interface propensities larger than the average propensity value are colored blue, the residues interacting with RNA is colored red, those residues colored yellow are the overlaps of the residues colored blue and red. All of the RNA molecules are colored orange

To illustrate the significance of the triplet interface propensity in the inference of interface residues, the residues of two proteins are colored from white to blue according to the propensity values, as shown in Fig. 3. Because the interface propensity of a certain residue triplet is represented by a 4D vector representing the four subtypes of the residue triplet, as mentioned in Methods section, we summarized the four values of the vector and used the results to color the center residue of the residue triplet. Larger calculated values are represented by darker colored residues. Figure 3(a) and (c) demonstrates that in most cases, the residues interacting with RNA are colored darker than those that do not interact, indicating that the feature triplet interface propensity may be distinguishing. The variation range of the summarized triplet interface propensities of residues in RBP195 was from −0.0149 to 3.1023; the mean triplet interface propensity of all of the protein residues in RBP195 was 0.1042. We hypothesize that the residues with summarized triplet interface propensities greater than the mean value are more likely to be RBRs; these residues are colored blue, while the others are colored white. According to our statistics on two proteins in Fig. 3 (b) and (d), the overlapping residues (colored yellow) between real RBRs (colored by red) and the blue- colored residues account for approximately 44.8 % of the real RBRs for chain A of protein 1QTQ, and the overlapping ratio is 47.3 % for chain A of protein 2ZZM. As shown in Fig. 3(b) and (d), many blue-colored residues are in the overlapping area; and these overlapping residues scatter in the protein-RNA interfaces of the two proteins.

**Fig. 4:**
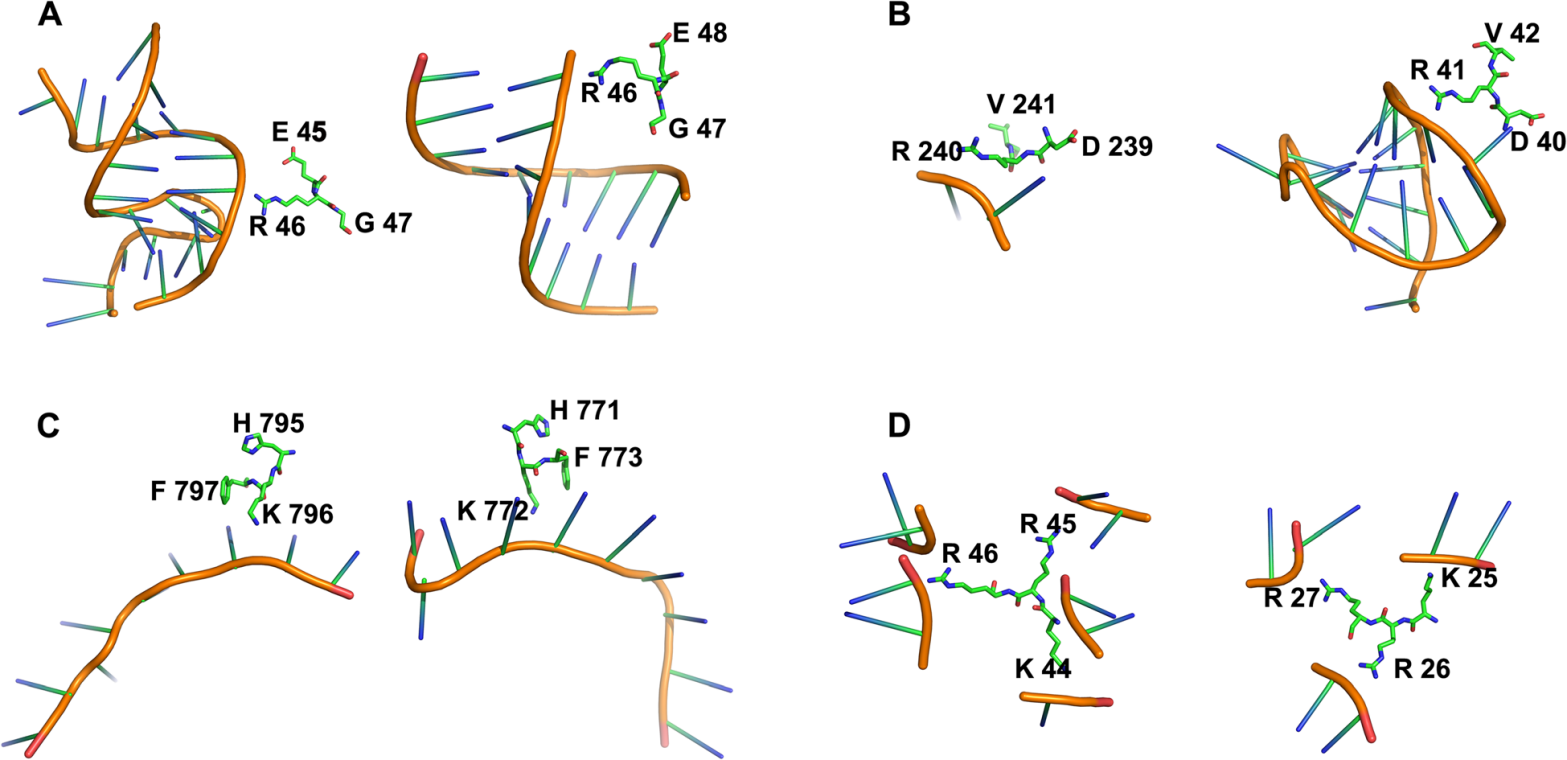
Interactions between RNAs and four types of interface triplets in different proteins. The two interface triplets in (**a**) are same as ERG and the first is from protein 2HVY_D, the second is from 2ZIO_A. The two interface triplets in (**b**) are same as DRV (the first and the second interface triplets are from 4LGT_A and 4GOA_A, respectively). In (**c**), two interface triplets HKF are from 3BX2_A and 3K49_A, respectively. The two interface triplets in (**d**) are same as KRR and the first is from 1VQ4_1, the second is from 2ZIO_A. The main chains of RNAs are colored orange

To demonstrate the necessity of describing the interface propensity of a residue triplet with a 4D vector, we explored all of the residue interface triplets on the protein-RNA interfaces in RBP195 and RBP68 and found that some of the interface residue triplets consisted of only one subtype of triplet_1, triplet_2, triplet_3 and triplet_4 (as described “Methods” section), and these interface triplets consisting of only subtype triplet_1, triplet_2, triplet_3 or triplet_4 accounted for approximately 8.37, 10.46, 11.15 and 36.03 % of all of the interface triplets, respectively. The other interface residue triplets consisted of more than one type of the four triplet types. The difference in the triplet subtypes of each type of residue interface triplets suggested that characterizing a given three-residue segment with a 4D vector could be meaningful for inferring the two neighboring residues of the centre residue. As shown in Fig. 4, four types of interface residue triplets consisting of only one of the four triplet subtypes with a relatively high interface propensity were used for this analysis. The first residue triplet is ERG, an interface triplet consisting of only triplet_1 in chain D of protein 2HVY and chain A of protein 2ZIO, in which the central arginine residue is bound to RNA using its long positively charged side chain, but the two neighboring residues, glycine and negatively charged glutamic acid, do not participate in RNA-protein interactions. In a triplet consisting of only triplet_2, the residue triplet DRV has its centre residue and the first residue of the triplet in the protein-RNA interface, as shown in chain A of proteins 4LGT and 4GOA. In ERG, the side chain of arginine is bound to the negatively charged phosphodiester backbone of RNA, but the negatively charged aspartic acid appears to bind to the nucleic acid base instead of the phosphodiester backbone because of electrostatic repulsion. The residue triplets HKF and KRR have only triplet_3 and triplet_4, respectively. In triplet HKF of chain A of proteins 3BX2 and 3 K49, the central residue and third residue of the triplet are RBRs. In the KRR of chain 1 of protein 1VQ4 and chain X of protein 4KIX, the three residues of the triplet are interface residues. In Fig. 4 (c) the conformations of the two triplets HKF have some similarities, and for the interface triplets KRR shown in Fig. 4 (d), the RNA residues interacting with the triplets KRR were frequently not consecutive along the sequences.

### Model construction using five types of calculated features

**Fig. 5:**
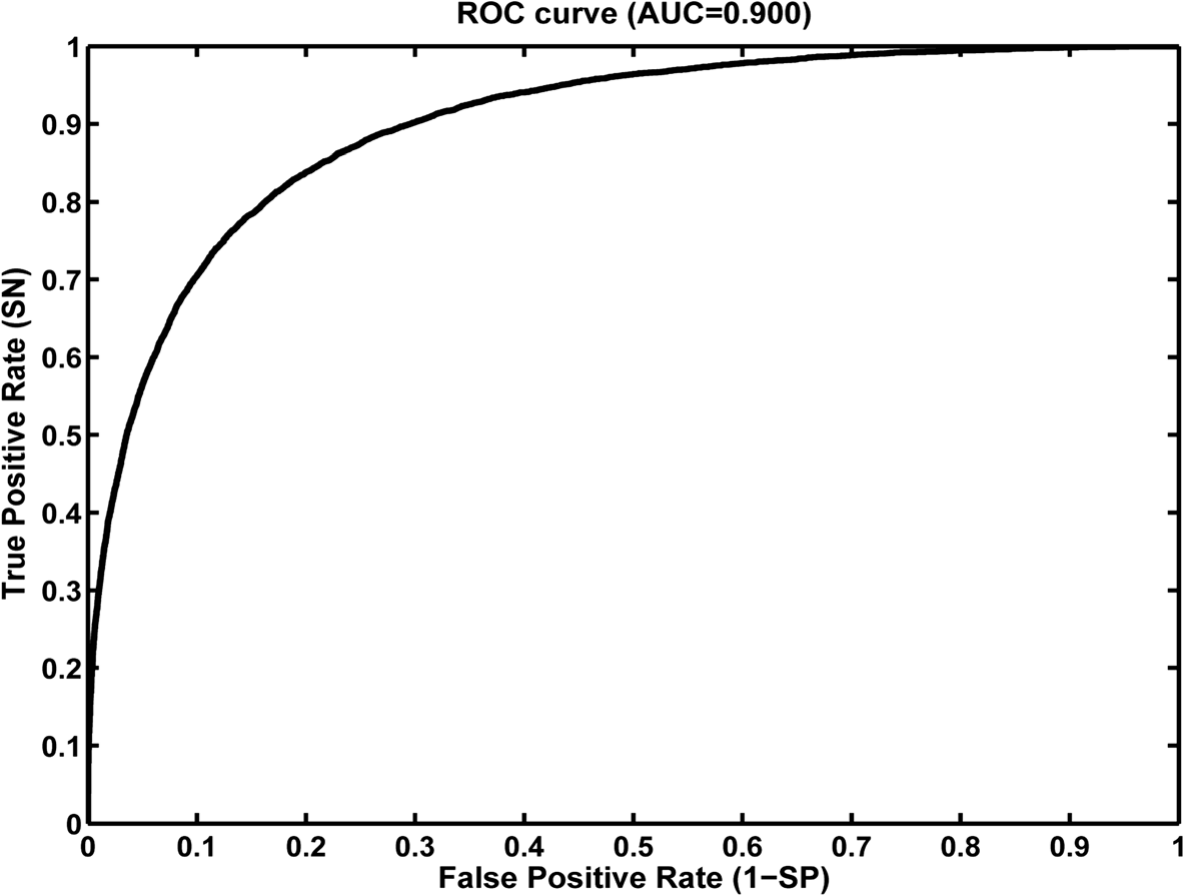
The ROC curve of five-fold cross-validation for our method on RBP195

In most cases, the background residues of a target residue are selected with the same patch type and patch size; here, we adopted the optimal patch type and patch size for each individual feature to select the background residues of the target residue. Thus, each chain is encoded by a feature vector of L*281 (see “Encoding scheme” section of “Methods”), where *L* represents the length of the protein chain. Based on the 195 protein chains in RBP195, we developed a prediction model using an RF classifier combined with the five types of characteristics using default parameters for RF algorithm, and to evaluate the robustness of our prediction model, a fivefold cross-validation was performed on RBP195. The ROC curve of the fivefold cross-validation is shown in Fig. 5. The prediction results of fivefold cross-validation when selecting other parameters of RF algorithm could be seen in Additional file 5. According to the AUC value of 0.900 for the ROC curve, we could conclude that the adoption of different structural and sequential features will help to develop a prediction model with good prediction performance.

### The contributions of each feature of RNAProSite

Five types of features are used to represent each residue in this study, to verify the effect of these five types of features for the predicting power of constructed prediction model, we extracted each type of feature from the whole feature vector and used the left four types of features to develop a RF classifier. Table 2 presents the results of fivefold validation on RBP198 for each developed RF classifier and we could find that the prediction performance will decrease when any type of feature is not adopted relative to that when all the five types of features are used. We could also find from Table 2 that the prediction performance decreases larger when triplet interface propensity was excluded from the whole feature vector than that when other types of features were extracted, which shows that the feature triplet interface propensity can provide more useful information concerning protein-RNA interaction. When comparing the results in Tables 3 and 2, we could find that although the prediction power of geometrical characteristic is relatively lower when used individually than other features, but excluding this feature can decrease the prediction performance larger than other features except triplet interface propensity, which shows that adopting a feature of different type will help to improve the prediction performance of the classifier when other discriminative features are used. From the importance values (mean decrease in accuracy and mean decrease in Gini index in Additional file 6) for each of the five types features, calculated by random forest algorithm when model construction, also proved that the two new structural features (electrostatic feature and triplet interface propensity) is helpful for the prediction of RNA-binding residues.

**Table 2:** The prediction performance for five-fold cross validation on RBP195 when one of the five types of features is excluded

Feature excluded	SN	SP	PPV	ACC	F-score	MCC
Electrostatic feature	0.700	0.894	0.530	0.865	0.606	0.533
Triplet interface propensity	0.669	0.862	0.458	0.833	0.544	0.458
PSSM profile	0.694	0.885	0.512	0.857	0.589	0.513
Geometrical characteristic	0.693	0.877	0.496	0.850	0.579	0.501
Physicochemical property	0.718	0.886	0.532	0.861	0.606	0.534
No feature excluded	0.720	0.892	0.550	0.867	0.616	0.546

**Table 3:** The prediction performance of five-fold cross validation for each individual feature on RBP195

Feature type	SN	SP	PPV	ACC	F-score	MCC
Electrostatic feature	0.490	0.865	0.388	0.809	0.433	0.323
Triplet interface propensity	0.565	0.924	0.564	0.871	0.565	0.489
PSSM profile	0.523	0.874	0.419	0.822	0.465	0.363
Geometrical characteristic	0.590	0.711	0.262	0.693	0.363	0.227
Physicochemical property	0.467	0.808	0.298	0.758	0.364	0.232

### Benchmark of prediction with RNAProSite and other excellent methods

To further evaluate the prediction performance of our prediction model, the non-redundant dataset RBP68 and RBP42 were used as comparative benchmark datasets. The cutoff value of 3.5 Å is used for the definition of RBRs for RBP42 and RBP138,the training dataset is RBP138 for our method when RBP42 is used as the benchmark dataset. Six kinds of available web-servers and one program are used, namely, BindN [24], Pprint [17], RNABindR [25], KYG [33], aaRNA [35], RBscore [36], PRNA [26]. We predicted the protein chains in RBP68 and RBP42 by our predictor and compared the prediction results with seven common available sequence-based and structure-based web servers in Table 4. As shown in Table 4, when tested on RBP68, the prediction sensitivity of RNAProSite was 0.707, which is better than all of the listed web servers except RNABindR [25] and RBscore [36], whose prediction sensitivity was 0.774 and 0.741, and the specificity, accuracy, positive predictive value, F-score and MCC of RNAProSite was better than the other prediction models on RBP68. We could find that when the cutoff value is set to 3.5 Å instead of 5 Å for RBP42, the prediction performance for all the methods mentioned in Table 4 decreased especially for predictors using structural features, the reason may be that less RBRs are defined and several of these non-RBRs with similar properties with RBRs are predicted as RBRs.

**Table 4:** The comparison of prediction performance between RNAProSite and other excellent sequence-based and structure-based methods on RBP68 (RBP42)

Methods	SN	SP	PPV	ACC	F-score	MCC
BindN (sn)	0.606 (0.600)	0.412 (0.417)	0.163 (0.093)	0.443 (0.433)	0.257 (0.160)	0.014 (0.009)
BindN (sp)	0.366 (0.388)	0.659 (0.667)	0.169 (0.1036)	0.613 (0.642)	0.231 (0.164)	0.019 (0.033)
Pprint	0.690 (0.673)	0.800 (0.780)	0.394 (0.233)	0.782 (0.770)	0.502 (0.346)	0.400 (0.296)
RNABindR	0.774 (0.805)	0.734 (0.728)	0.351 (0.227)	0.740 (0.735)	0.483 (0.354)	0.388 (0.327)
KYG	0.550 (0.605)	0.813 (0.793)	0.357 (0.225)	0.771 (0.776)	0.432 (0.328)	0.308 (0.266)
aaRNA	0.645 (0.695)	0.882 (0.863)	0.510 (0.335)	0.845 (0.848)	0.569 (0.452)	0.481 (0.410)
PRNA	0.617 (0.592)	0.719 (0.673)	0.294 (0.255)	0.703 (0.660)	0.398 (0.356)	0.261 (0.201)
RBScore	0.741 (0.675)	0.876 (0.860)	0.530 (0.476)	0.854 (0.830)	0.618 (0.558)	0.542 (0.468)
RNAProSite	0.707 (0.665)	0.900 (0.914)	0.606 (0.434)	0.868 (0.892)	0.631 (0.525)	0.557 (0.481)

### The effects of dataset, algorithm, and the definition of RBRs on the prediction performance

It is known that if the sequences or structures in a dataset have some kind of similarities, then the classifier may learn these similarities and result a prediction model with relatively better performance than expected, so we compared the prediction result of five-fold cross validation on RB195 and RB138. From the results of “5 Å (RB195, RF)” and “5 Å (RB138, RF)” in Table 5, we could find that the values of SN for RB195 is only slightly higher RBP138, but the SP, ACC, F-score, MCC and PPV for RB195 is slightly lower than that for RBP138, which means that our method seems not sensitive to the composition of dataset. We could find that the different machine learning algorithms may have different five-fold cross validation results from the prediction results of “5 Å (RB138, RF)” and “5 Å (RB138, SVM)” and the random forest classifier was better than the SVM classifier when the features in our method were adopted, which could also be found when analyze the results of “3.5 Å (RB138, RF)” and “3.5 Å (RB138, SVM)”. For the construction of SVM classifier, the kernel function of RBF was used and other parameters are optimized by grid search method to deliver high accuracy. When comparing the results of “5 Å (RB138, RF)” and “3.5 Å (RB138, RF)”, we could find that the prediction results is slightly worse considering the balanced measures of F-score and MCC when the cutoff value is set to a lower value of 3.5 Å relative to that when a cutoff value of 5 Å is chosen, this phenomenon could also be found in the study of comparison for different prediction methods [58]. From the recently published study [59] for comparing the performance of different prediction methods, we could also find that our method shows stable prediction performance when different distance cutoff values and datasets are chosen.

**Table 5:** The effects of datasets, algorithm, and the definition of RBRs on the prediction performance of our method

Cutoff (Dataset, algorithm)	SN	SP	PPV	ACC	F-score	MCC
5 Å (RB195, RF)	0.720	0.892	0.550	0.867	0.616	0.546
5 Å (RB138, RF)	0.678	0.910	0.568	0.876	0.618	0.547
5 Å (RB138, SVM)	0.621	0.900	0.531	0.858	0.566	0.485
3.5 Å (RB138, RF)	0.670	0.937	0.525	0.912	0.588	0.545
3.5 Å (RB138, SVM)	0.630	0.908	0.415	0.882	0.500	0.449

### The effects of conformational change upon binding RNA

To assess whether the performance of RNAProSite would be affected by protein conformational changes that accompany RNA binding, we used the 35 RNA-free structures and 35 respective RNA-bound structure from a published dataset DatasetII [35],the root-mean-square deviation (RMSD) of the C_α_ atoms for each pair of RNA-free and respective RNA-bound structures ranges from 0.35 to 8.87 Å. From the results in Table 6 we could find that the prediction results for RNA-bound proteins are only slightly better than that for RNA-free protein and the difference values of the six evaluation measures between RNA-bound proteins and RNA-free proteins are not more than 0.06. By analyzing the results in Table 7 we could find that the RMSD values for most pairs of RNA-free and respective RNA-bound proteins are between 1 Å to 2 Å. For seven pairs of these proteins whose RMSD distribution intervals are in “[2 Å, 3 Å)” and “[3 Å, 4 Å)”, the prediction performance for RNA-free proteins decreases more relative to that for RNA-bound proteins when comparing to proteins in other RMSD distribution intervals in Table 7. In a whole, our method is not very sensitive to the conformational changes upon RNA binding.

**Table 6:** The effects of conformational change upon RNA binding on the prediction performance of our methods

Protein type	SN	SP	PPV	ACC	F-score	MCC
RNA-free	0.778	0.845	0.419	0.837	0.545	0.488
RNA-bound	0.810	0.865	0.474	0.858	0.598	0.546

**Table 7:** The pairs of RNA-bounding and RNA-free proteins and performance differences between two types of proteins in different intervals of RMSD distribution (D_SN, D_SP, D_PPV, D_ACC, D_F_score and D_MCC stand for the prediction performance decrease in SN, SP, PPV, ACC, F_score and MCC for RNA-free proteins relative to that for RNA-bound ones)

RMSD distribution	Pairs	D_SN	D_SP	D_PPV	D_ACC	D_F_score	D_MCC
[0 Å, 1 Å)	7	0.039	0.023	0.046	0.024	0.051	0.058
[1 Å, 2 Å)	16	−0.040	0.060	0.099	0.048	0.079	0.069
[2 Å, 3 Å)	4	0.012	0.071	0.120	0.059	0.086	0.108
[3 Å, 4 Å)	3	0.125	0.029	0.067	0.036	0.090	0.112
[4 Å, 9 Å)	5	−0.008	0.037	0.072	0.027	0.048	0.051

## Discussion

**Fig. 6:**
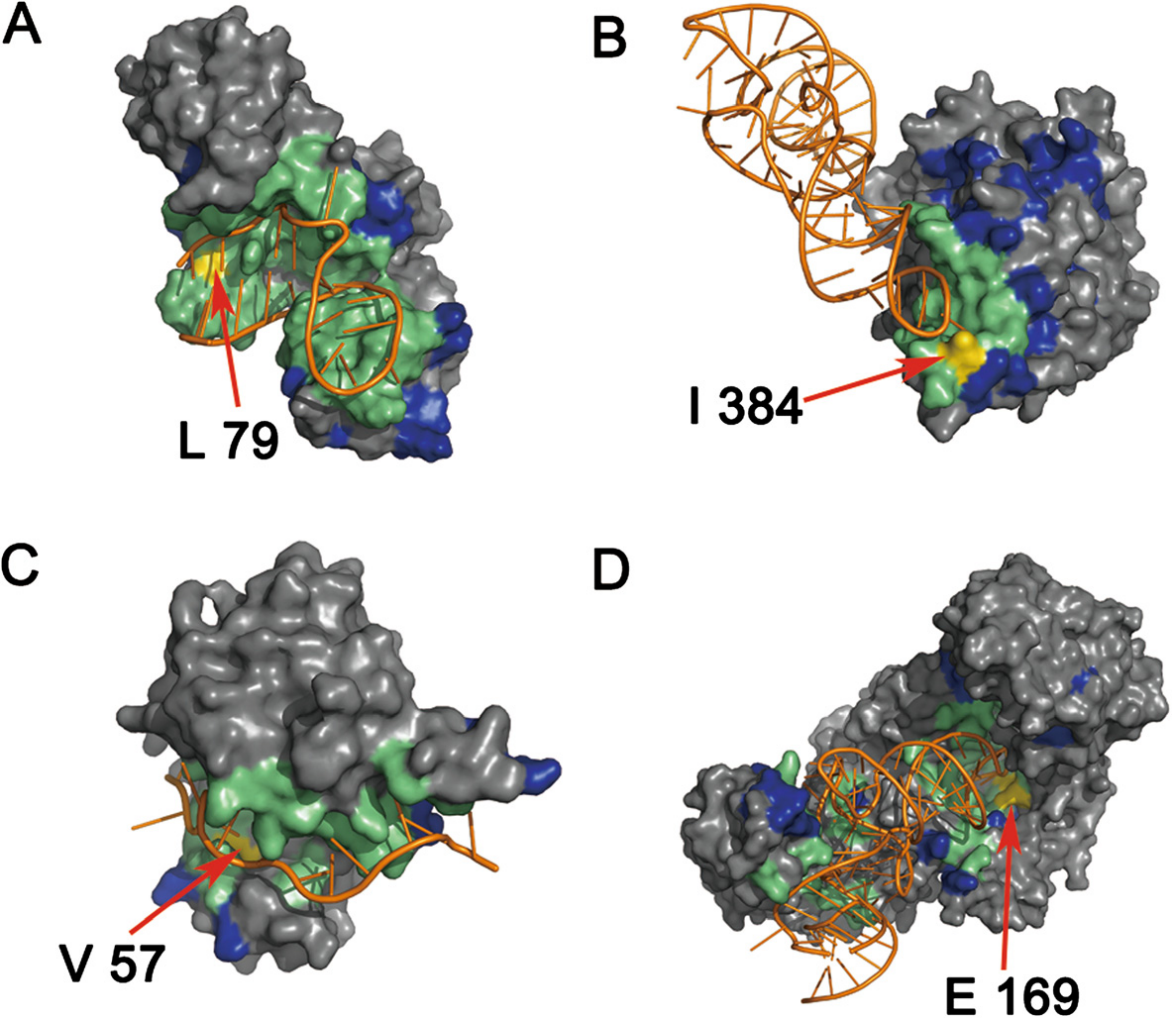
The prediction results of RNAProSite on four RBP chains. A residue is colored blue when it is falsely predicted as RNA-binding and green when it is truly predicted as RNA-binding. The residues colored by yellow mean they are truly predicted as RBRs but not predicted by other methods. The RNA is colored orange. The PDB codes of the four RBP chain in (**a**), (**b**), (**c**) and (**d**) are 4GLT (chain A), 2AZX (chain A), 3QJJ (chain A) and 3ZGZ (chain A), respectively

Due to the methodological differences, RNAProSite may identify some real RBRs that cannot be determined by the other seven approaches. So we selected four protein chains from RBP68 and searched some of the residues predicted by RNAProSite but not by the other seven common methods (Except the glutamic acid in Fig. 6(d) that was also truly predicted by RBscore), as shown in Fig. 6. According to the RBRs predicted by RNAProSite but rarely by the other methods, all of the yellow-colored residues had positive electrostatic surface potential values and were in the largest positive surface patch, except for the residue in Fig. 6(d). We selected the RBP chain 3ZGZ (chain A) to analyze the important contributions of the triplet interface propensity feature in prediction of RBRs, Because the yellow-colored glutamic acid in 3ZGZ (chain A) had a negative charge and was in the largest negative surface path and because glutamic acid is rarely located on the RNA-protein interface relative to other positive-charged residues. The surface residue triplet of the residue glutamic is NEQ, this triplet contains only the second type of triplet_1, triplet_2, triplet_3 and triplet_4 (see ‘Methods’ section), and its propensity value is 0.1252, which is higher than the mean 0.0251 of all analyzed triplets, meaning that the first and centre residues may be RNA-binding instead of the third residue for the surface triplet NEQ (only predicted by RBscore and RNAProSite). According to our statistics on the prediction results of our methods and other seven prediction programs in Additional file 7, we could find that each prediction method could find really RNA-binding residues not predicted by other prediction methods, which proved the difference in adopted features for predicting RNA-binding sites on proteins may lead to the difference in prediction results.

## Conclusion

In this study, we designed two discriminative structure-derived features, namely residue electrostatic surface potential and triplet interface propensity, to characterize a protein residue together with other commonly used descriptors. A comprehensive analysis of the two newly designed features from different aspects demonstrated that the two features have excellent discriminative power on a large dataset and may reflect the underlying mechanisms of RNA-protein interactions. To incorporate information from neighbor residues to determine the RNA-binding properties of each target residue, the optimal patch type and patch size for different features are searched, and by using the searched optimal patch type and patch size for each used feature, a random forest classifier is developed and implemented in the web server RNAProSite. From the results of a fivefold cross validation on a training set and the prediction performance on a test set, we concluded that our method can predict RBRs with results better than or comparable to those of the existing approaches and could assist researchers in performing more targeted assays.

## Abbreviations

ACC, accuracy; AUC, area under the receiver operating characteristic curve; MCC, Matthews’s correlation coefficient; PPV, positive predictive value; RBPs, RNA Binding Proteins; RBRs, RNA-binding residues; SN, sensitivity; SP, specificity

## Additional files

Additional file 1: A complete list of all of the PDB codes for datasets RBP195, RBP68, RBP138 and RBP42. (DOC 40 kb) Additional file 2: The effect of RSA cutoff values on prediction performance for triplet interface propensity. (DOC 33 kb) Additional file 3: Details for the selection of physicochemical properties from AAIndex database. (DOC 31 kb) Additional file 4: The selection of optimal patch type and patch size for each type of feature. (DOC 34 kb) Additional file 5: The effects of number of trees grown (ntree) and number of predictors sampled (mtry) on prediction performance. (DOC 40 kb) Additional file 6: Statistics for the number of truly predicted RNA-binding residues (nTPs) only by one prediction model. (DOC 29 kb) Additional file 7: The mean decrease in accuracy (De_acc) and Gini index (De_Gini) for five types of features. (DOC 31 kb)
