# Supplementary material for: Accurate prediction of RNA-binding protein residues with two discriminative structural descriptors
Source: BMC Bioinformatics. 2016 Jun 7;17:231. doi: 10.1186/s12859-016-1110-x (PMC4897909; doi:10.1186/s12859-016-1110-x)
Supplement: Additional file 1: — A complete list of all of the PDB codes for datasets RBP195, RBP68, RBP138 and RBP42. (DOC 40 kb) [file 12859_2016_1110_MOESM1_ESM.doc]

A complete list of all of the PDB codes for datasets RBP195, RBP68, RBP138 and RBP42

| **List of protein chains in RBP195** | | | | | |
| --- | --- | --- | --- | --- | --- |
| 3AHU_A  2J01_5  1GAX_A  2R7R_A  4B3G_A  1YIT_H  3EQT_A  1FJG_Q  1VQ4_Y  4AL7_A  3G0H_A  3RW6_A  2RD2_A  2GXB_A  1MZP_A  4KIY_F  1YYK_A  4M7A_C  1Q2R_A  2GJW_A  2X1A_A  2XNR_A  3CMA_Z  2ZI0_A  1H4Q_A  3RC8_A  3IE1_A  2G4B_A  3R8S_R  2J01_3  3KS8_A  3R2D_A  2ZJR_M  4KIY_N | 3ADB_A  3D2S_A  4C7O_B  3IAB_B  1L9A_A  1VQ4_A  2R8S_L  3DD2_H  3F1F_I  2ZJR_T  2A8V_A  2AZ0_A  3IAB_A  3SN2_A  3ER9_B  4E78_A  3AEV_B  1H2D_A  1N8R_L  3KFU_A  4KIY_U  3OUY_A  3EPH_A  1FJG_C  2XGJ_A  3VYY_A  3R8S_Y  2I82_A  2ASB_A  3U2E_A  1FJG_F  1SER_A  3A6P_A  4KIY_E | 1JID_A  1B23_P  2ZJR_R  4HOR_A  2DU3_A  1FJG_P  1VQ4_2  4GD1_P  1YVP_A  2ZJR_S  2R8S_H  2ZJR_J  2J01_C  2CSX_A  1VQ4_P  1FJG_R  4C8Y_A  1WMQ_A  4KIX_X  1VQ4_Q  3TRZ_A  4J7L_A  4IFD_B  3V2C_Y  1ZH5_A  3ADL_A  4ERD_A  3KFU_E  2J01_F  1VQ4_1  2ZJR_I  3IEV_A  1AQ3_A  4II9_A | 4KIY_V  4KIX_N  2J01_7  2HYI_C  4BPB_A  2HVY_A  2HVY_C  4L8R_C  3NMR_A  3R8S_H  3GPQ_A  2ANN_A  3CMA_M  1M5K_C  4M7A_A  2J01_U  2QUX_A  4M4O_A  4C9D_A  3B0U_X  1WNE_A  1DFU_P  2J01_D  3K49_A  2DB3_A  3O3I_X  1IL2_A  1VQ4_V  1I6U_A  2ZKO_A  3M7N_D | 2GIC_A  1N8R_E  1BMV_2  1EC6_A  3SQW_A  1FFY_A  1KNZ_A  3HL2_A  1M8V_A  2ZJR_H  2FMT_A  1N8R_K  1J1U_A  2PJP_A  4K4X_A  1VQ4_X  4IFD_J  4L8H_A  1JBR_A  3OIN_A  3AKZ_A  4IFD_H  3ZC0_A  2OZB_B  1N8R_X  4KIX_3  3F1E_X  3MDG_A  3T5N_A  1FJG_B  1VQ4_N | 1VQ4_E  1A34_A  1FJG_M  2PLY_A  3NMU_A  4KIX_0  1VQ4_D  1VQ4_O  3MOJ_B  4KIX_T  4KIX_5  4L8R_B  1R3E_A  4KXT_A  3QRP_A  4NKU_A  4KIY_I  1HQ1_A  3AMT_A  1FJG_K  1FJG_J  2IX1_A  1MJI_A  4IFD_A  1U0B_B  1VQ4_I  1YTU_A  2PY9_A  2ZJR_E  3VYX_A  3QG9_A |
| **List of protein chains in RBP68** | | | | | |
| 2ZJR_P  4KIY_T  2BH2_A  4N2Q_A  4F02_A  4M7A_G  2AZX_A  1VQ4_T  4M59_A  3T3O_A  2OZB_A  1K8W_A | 2J01_1  3NDB_B  4KIY_G  4ED5_A  2Q66_A  1F7Y_A  1FJG_L  4IFD_G  2F8K_A  1OOA_A  4G0A_A  3ZGZ_A | 4H5P_A  2JLU_A  1N8R_I  3H5X_A  2ZZM_A  2FK6_A  1FJG_S  3RTJ_A  3AGV_A  2ZJR_B  1FJG_D  1VQ4_B | 1UVI_A  3WBM_A  3FTE_A  4M7A_B  3CMA_U  1F7U_A  3R8S_E  2XS2_A  3G9Y_A  1VQ4_R  2B3J_A | 2J01_2  3DH3_A  4J1G_A  3BX2_A  1FJG_H  2JEA_A  3R8S_4  2F8S_A  1N8R_4  1N1H_A  1QF6_A | 1RPU_A  1FEU_A  4KIX_O  3QJJ_A  1FJG_T  4LGT_A  2ZM5_A  1VQ4_L  4FVU_A  2ZJR_G |
| **List of protein chains in RBP138** | | | | | |
| 3AHU_A  4KIY_V  2GIC_A  2J01_5  3D2S_A  4KIX_N  1N8R_E  1A34_A  1GAX_A  2ZJR_R  2J01_7  1BMV_2  1FJG_M  2R7R_A  3IAB_B  4HOR_A  2PLY_A  4B3G_A  1L9A_A  2DU3_A  4BPB_A  3SQW_A  3NMU_A | 1YIT_H  1KNZ_A  1VQ4_D  3DD2_H  1VQ4_2  2HVY_C  3HL2_A  1VQ4_O  1VQ4_Y  4GD1_P  4L8R_C  3MOJ_B  4AL7_A  2ZJR_T  1YVP_A  2ZJR_H  4KIX_T  2ZJR_S  3R8S_H  2FMT_A  4KIX_5  2AZ0_A  3GPQ_A | 4L8R_B  3IAB_A  2ZJR_J  2GXB_A  3SN2_A  3CMA_M  4KXT_A  1MZP_A  3ER9_B  3QRP_A  4KIY_F  4E78_A  1VQ4_P  1VQ4_X  4NKU_A  3AEV_B  1FJG_R  4M7A_A  4IFD_J  4KIY_I  4M7A_C  1H2D_A  2J01_U | 1Q2R_A  2QUX_A  4L8H_A  3AMT_A  2GJW_A  3KFU_A  1WMQ_A  4M4O_A  1JBR_A  1FJG_K  4KIY_U  4KIX_X  4C9D_A  3OIN_A  1FJG_J  3OUY_A  1VQ4_Q  3B0U_X  3AKZ_A  2IX1_A  3CMA_Z  3EPH_A  3TRZ_A | 1WNE_A  4IFD_H  2ZI0_A  4J7L_A  3ZC0_A  4IFD_A  2XGJ_A  2J01_D  3RC8_A  3VYY_A  3V2C_Y  3K49_A  1N8R_X  1VQ4_I  3IE1_A  4KIX_3  2I82_A  3ADL_A  3O3I_X  3F1E_X  2PY9_A  3R8S_R  4ERD_A | 2ZJR_E  2J01_3  3U2E_A  3KFU_E  1VQ4_V  3T5N_A  3VYX_A  3KS8_A  2J01_F  1I6U_A  1FJG_B  3QG9_A  3R2D_A  2ZKO_A  1VQ4_N  4II9_A  2ZJR_M  3A6P_A  2ZJR_I  3M7N_D  3IEV_A  4KIY_N  4KIY_E |
| **List of protein chains in RBP42** | | | | | |
| 2ZJR_P  2J01_1  4H5P_A  1UVI_A  2J01_2  1RPU_A  3NDB_B | 2JLU_A  3WBM_A  4KIY_G  1N8R_I  3FTE_A  4J1G_A  3H5X_A | 4M7A_B  3QJJ_A  4F02_A  2ZZM_A  3CMA_U  1FJG_T  4LGT_A | 1FJG_S  3R8S_4  1VQ4_T  4IFD_G  3RTJ_A  2F8S_A  4M59_A | 2F8K_A  3AGV_A  3G9Y_A  1N8R_4  4FVU_A  3T3O_A  1N1H_A | 2ZJR_G  2OZB_A  4G0A_A  1FJG_D  2B3J_A  3ZGZ_A  1VQ4_B |
