# Supplementary material for: Accurate prediction of RNA-binding protein residues with two discriminative structural descriptors
Source: BMC Bioinformatics. 2016 Jun 7;17:231. doi: 10.1186/s12859-016-1110-x (PMC4897909; doi:10.1186/s12859-016-1110-x)
Supplement: Additional file 2: — The effect of RSA cutoff values on prediction performance for triplet interface propensity. (DOC 33 kb) [file 12859_2016_1110_MOESM2_ESM.doc]

The effect of RSA cutoff values on prediction performance for triplet interface propensity.

| RSA cutoff | SN | SP | MCC | PPV | ACC | F-score |
| --- | --- | --- | --- | --- | --- | --- |
| 1% | 0.547 | 0.922 | 0.471 | 0.551 | 0.867 | 0.549 |
| 3% | 0.565 | 0.924 | 0.489 | 0.564 | 0.871 | 0.565 |
| 5% | 0.532 | 0.919 | 0.451 | 0.533 | 0.861 | 0.532 |
| 7% | 0.524 | 0.918 | 0.441 | 0.526 | 0.859 | 0.524 |
| 9% | 0.514 | 0.916 | 0.430 | 0.516 | 0.856 | 0.515 |
