# Supplementary material for: Accurate prediction of RNA-binding protein residues with two discriminative structural descriptors
Source: BMC Bioinformatics. 2016 Jun 7;17:231. doi: 10.1186/s12859-016-1110-x (PMC4897909; doi:10.1186/s12859-016-1110-x)
Supplement: Additional file 3: — Details for the selection of physicochemical properties from AAIndex database. (DOC 31 kb) [file 12859_2016_1110_MOESM3_ESM.doc]

**Details for the selection of physicochemical properties from AAIndex database**

To select the physicochemical properties with a relatively stronger predictive power in the AAIndex, we firstly measured the correlation coefficient ($r=\sum_{i=1}^{i=20} (x_{i}-\bar{x})(y_{i}-\bar{y})/\sqrt{\sum_{i=1}^{i=20} (x_{i}-\bar{x})^{2}\sum_{i=1}^{i=20} (y_{i}-\bar{y})^{2}}$) between twenty values of each property in the AAIndex and that of interface propensities ($P_{k}$) for twenty types of amino acid residues, where $x_{i}$ and $y_{i}$ represent the value of two types of properties for amino acid type , and $\bar{x}$ and $\bar{y}$ are the mean of twenty values for twenty types of amino acid of the two types of properties. This type of correlation coefficient may not fully reflect the predictive power of these amino acid indices but is an alternative method for feature selection. The properties with the highest absolute correlation coefficient values were selected. Then, we calculated the absolute values of correlation coefficient between each physicochemical property, and for each likely pair of properties with correlation coefficient more than 0.8, we discarded the properties with lower correlation coefficient with interface propensities ($P_{k}$). Thus, ten properties were selected.
