# Supplementary material for: Accurate prediction of RNA-binding protein residues with two discriminative structural descriptors
Source: BMC Bioinformatics. 2016 Jun 7;17:231. doi: 10.1186/s12859-016-1110-x (PMC4897909; doi:10.1186/s12859-016-1110-x)
Supplement: Additional file 4: — The selection of optimal patch type and patch size for each type of feature. (DOC 34 kb) [file 12859_2016_1110_MOESM4_ESM.doc]

**The selection of optimal patch type and patch size for each type of feature**

To obtain the optimal patch size and patch type for a given sequential or structural feature, we first encoded each protein residue with each individual feature using a sequential patch and structural patch, respectively. The patch size selected for the sequential and structural patches ranged from one to thirteen, with a step size of two. Then, we performed a fivefold cross validation on RBP195 for each combination of patch type and patch size to determine the optimal patch type and patch size for each feature when the F-score for this type of combination was greater than that of the other combinations. For each fivefold cross validation result, a series of F-score values was computed by using a range of cut-off values from -1 to 1 with a step size of 0.001, and the best value was selected as the F-score for this five-fold cross validation. To avoid data dimensions that were too large and excessively noisy data, we decided that the patch size was optimal for a particular patch type when the F-score increases less than 0.01 when increasing the patch size. As could be seen in Table S1, the optimal patch type for all the five types of features except electrostatic feature is sequential patch, the optimal patch size for electrostatic feature, triplet interface propensity, PSSM profile, geometrical characteristic and physicochemical property is 11, 7, 5,5 and 9, respectively.

**Table S1**. The optimal patch type and patch size for five types of features.

| Feature | Sequential patch | | | Structural patch | | |
| --- | --- | --- | --- | --- | --- | --- |
|  | Optimal patch size | The best F-score | AUC | Optimal patch size | The best F-score | AUC |
| Electrostatic feature | 9 | 0.429 | 0.756 | 11 | 0.433 | 0.762 |
| Triplet interface propensity | 7 | 0.565 | 0.837 | 7 | 0.504 | 0.816 |
| PSSM profile | 5 | 0.465 | 0.795 | 7 | 0.439 | 0.780 |
| Geometrical characteristic | 5 | 0.363 | 0.728 | 7 | 0.355 | 0.717 |
| Physicochemical property | 9 | 0.364 | 0.699 | 9 | 0.351 | 0.681 |
