# Supplementary material for: Accurate prediction of RNA-binding protein residues with two discriminative structural descriptors
Source: BMC Bioinformatics. 2016 Jun 7;17:231. doi: 10.1186/s12859-016-1110-x (PMC4897909; doi:10.1186/s12859-016-1110-x)
Supplement: Additional file 5: — The effects of number of trees grown (ntree) and number of predictors sampled (mtry) on prediction performance. (DOC 40 kb) [file 12859_2016_1110_MOESM5_ESM.doc]

The effects of number of trees grown (ntree) and number of predictors sampled (mtry) on prediction performance (default values of ntree and mtry in this study is 500 and 18, individually).

| ntree, mtry | SN | SP | MCC | PPV | ACC | F-score |
| --- | --- | --- | --- | --- | --- | --- |
| 100, 18 | 0.463 | 0.934 | 0.436 | 0.576 | 0.861 | 0.513 |
| 200, 18 | 0.476 | 0.920 | 0.433 | 0.559 | 0.857 | 0.514 |
| 300, 18 | 0.527 | 0.938 | 0.497 | 0.617 | 0.873 | 0.569 |
| 400, 18 | 0.563 | 0.936 | 0.522 | 0.620 | 0.877 | 0.592 |
| **500, 18** | **0.562** | **0.933** | **0.512** | **0.612** | **0.874** | **0.586** |
| 600, 18 | 0.554 | 0.936 | 0.514 | 0.621 | 0.876 | 0.586 |
| 500, 6 | 0.398 | 0.949 | 0.412 | 0.596 | 0.861 | 0.478 |
| 500, 10 | 0.502 | 0.942 | 0.486 | 0.622 | 0.872 | 0.555 |
| 500, 14 | 0.538 | 0.934 | 0.497 | 0.607 | 0.871 | 0.571 |
| 500, 22 | 0.544 | 0.937 | 0.508 | 0.621 | 0.875 | 0.580 |
| 500, 26 | 0.557 | 0.933 | 0.510 | 0.612 | 0.874 | 0.583 |
