# Supplementary material for: Accurate prediction of RNA-binding protein residues with two discriminative structural descriptors
Source: BMC Bioinformatics. 2016 Jun 7;17:231. doi: 10.1186/s12859-016-1110-x (PMC4897909; doi:10.1186/s12859-016-1110-x)
Supplement: Additional file 6: — Statistics for the number of truly predicted RNA-binding residues (nTPs) only by one prediction model. (DOC 29 kb) [file 12859_2016_1110_MOESM6_ESM.doc]

The mean decrease in accuracy (De_acc) and Gini index (De_Gini) for five types of features

| Feature | De_acc | De_Gini |
| --- | --- | --- |
| Electrostatic feature | 0.0008 | 74.98 |
| Triplet interface  propensity | 0.0016 | 90.63 |
| PSSM profile | 0.0007 | 50.25 |
| Geometrical characteristic | 0.0029 | 90.38 |
| Physicochemical property | 0.0002 | 30.26 |
