# Supplementary material for: Accurate prediction of RNA-binding protein residues with two discriminative structural descriptors
Source: BMC Bioinformatics. 2016 Jun 7;17:231. doi: 10.1186/s12859-016-1110-x (PMC4897909; doi:10.1186/s12859-016-1110-x)
Supplement: Additional file 7: — The mean decrease in accuracy (De_acc) and Gini index (De_Gini) for five types of features. (DOC 31 kb) [file 12859_2016_1110_MOESM7_ESM.doc]

Statistics for the number of truly predicted RNA-binding residues (nTPs) only by one prediction model.

| Methods | nTPs |
| --- | --- |
| BindN (sn) | 93 |
| BindN (sp) | 0 |
| Pprint | 79 |
| RNABindR | 37 |
| KYG | 44 |
| aaRNA | 44 |
| PRNA | 25 |
| RBScore | 29 |
| RNAProSite | 36 |
